# The rural tax: comprehensive out-of-pocket costs associated with patient travel in British Columbia

**DOI:** 10.1186/s12913-021-06833-2

**Published:** 2021-08-21

**Authors:** Jude Kornelsen, Asif Raza Khowaja, Gal Av-Gay, Eva Sullivan, Anshu Parajulee, Marjorie Dunnebacke, Dorothy Egan, Mickey Balas, Peggy Williamson

**Affiliations:** 1 Centre for Rural Health Research, Department of Family Practice, University of British Columbia, Suite 320 – 5950 University Blvd, Vancouver, BC V6T 1Z3 Canada; 2 Faculty of Applied Health Sciences, Brock University, Niagara Region, 1812 Sir Isaac Brock Way, St. Catharines, ON L2S 3A1 Canada

**Keywords:** Rural and remote health, Patient transportation, Out of pocket costs, Access to care, Barriers to health care, Health disparities

## Abstract

**Background:**

A significant concern for rural patients is the cost of travel outside of their community for specialist and diagnostic care. Often, these costs are transferred to patients and their families, who also experience stress associated with traveling for care. We sought to examine the rural patient experience by (1) estimating and categorizing the various out of pocket costs associated with traveling for healthcare and (2) describing and measuring patient stress and other experiences associated with traveling to seek care, specifically in relation to household income.

**Methods:**

We have designed and administered an online, retrospective, cross-sectional survey seeking to estimate the out-of-pocket (OOP) costs and personal experiences of rural patients associated with traveling to access health care in British Columbia. Respondents were surveyed across five categories: Distance Traveled and Transportation Costs, Accommodation Costs, Co-Traveler Costs, Lost Wages, and Patient Stress. Bivariate relationships between respondent household income and other numerical findings were investigated using one-way ANOVA.

**Results:**

On average, costs for respondents were $856 and $674 for transport and accommodation, respectively. Strong relationships were found to exist between the distance traveled and total transport costs, as well as between a patient’s stress and their household income. Patient perspectives obtained from this survey expressed several related issues, including the physical and psychosocial impacts of travel as well as delayed or diminished care seeking.

**Conclusions:**

These key findings highlight the existing inequities between rural and urban patient access to health care and how these inequities are exacerbated by a patient’s overall travel-distance and financial status. This study can directly inform policy related efforts towards mitigating the rural-urban gap in access to health care.

## Background

Despite service planning challenges (low population densities spread over vast geographies, seasonal inclement weather), jurisdictions across Canada have developed relatively robust infrastructures to affect emergency patient transport [1–3]. Likewise, most provinces and territories have established effective mechanisms for inter-facility (hospital to hospital) patient transport within regionalized care [4]. However, there are few safety nets to facilitate transport to care in non-urgent or consultative situations, and in most jurisdictions for most residents, these costs are borne by individuals and families. For example, in Saskatchewan (Canada), 72% of all surgeries are performed in Regina and Saskatoon. Lavis and Boyko found that many people who live outside of those urban centres may not have the option of traveling there for care, partly due to accommodation costs [5]. Likewise, in *Monitoring Seniors Services* (Office of the Seniors Advocate, 2018), the senior citizen advocate noted the importance of public transportation in maintaining seniors’ independence and optimal health, and emphasized the importance of services such as HandyDART (a shared door-to-door public transit services for people with disabilities) and province-wide community programs to support seniors to live independently and be mobile, such as *Better at Home* and *Volunteer Drivers* [6]. The authors also noted that reliance on non-subsidized transport (e.g., taxis) is not viable for many on a fixed income. Despite these policy imperatives, out-of-pocket transport costs to access health care remain substantial for many rural citizens and, in some instances, create barriers in access to care.

One ongoing challenge for rural health planning is regional travel for patients that require a higher level of care than what is available locally. This may be for episodic or chronic specialist care, diagnostics or returning from an acute event or planned surgical care. In these instances, travel costs fall outside the health care system’s responsibility, leading to transferred expenses for patients and their families. While this is not a concern in urban centres with ready access to specialist care, including surgery, and diagnostics, it is a challenge for many rural residents who face highly limited public transport options. This has the biggest impact on individuals without private vehicles, including those without the ability to drive such as children, elderly, and those with disabilities [7]. Out of pocket (OOP) costs include expenses for care that are not reimbursed by insurance, as well as patient-specific costs such as travel to the referral site, food, accommodation, and for some, co-traveler costs. The difficulties associated with unsupported travel are compounded for populations that lack the necessary financial and social resources. Poverty is often cited as a risk factor for health [8, 9]. Despite this challenge to the fundamental tenant of public health care (access), there is scant understanding of costs incurred for rural patients who need to leave their communities for care, leading to a de facto “rural tax” on health care for rural residents.

There is a growing recognition of the plurality of health care experiences across the urban-rural divide. Compared to urban residents, rural and remote Canadians “experience shorter life expectancy, higher mortality rates […], higher prevalence of risk factors for chronic illness, higher hospitalization rates, greater utilization of emergency room services, and less access to after-hours care” [10]. A rigorous exploration of the consequences of a lack of immediate access to health care has been primarily confined to a comparison of health outcomes with urban patients as well as the psychosocial aspect of a lack of access to care [11, 12]. In countries such as China and Australia where OOP costs for rural patients have been considered, there is evidence to show that financial considerations impact care-seeking behaviours. For example, in China, OOP costs have been shown to be “highly valued” in the decision-making process of patients on where to access care [13]. Similarly, studies in Australia have found that treatment decisions do appear to be shaped by conflict between treatment location, costs of traveling for care, disruption of life, as well as by the rigidity of available financial assistance policies [14–16]. Additionally, Lyford et al. highlighted difficulty arranging and undertaking travel in Western Australia as being one of the main reasons for poorer outcomes of Aboriginal people with cancer [17].

Telemedicine has been identified as one mechanism for reducing the burden of rural patient travel. For example, one study out of Texas on the use of telehealth to conduct pre-surgery evaluations for families of children with cerebral palsy estimated that over 3 years, the telemedicine pre-op screen saved 106,070 miles of transportation and resulted in savings of $55,326 USD [18]. A similar study examining urology care delivered via telehealth over a 6 month period found that patients saved an average of 277 travel miles, 290 min of travel time, $67 in travel expenses and $126 in lost opportunity cost [19]. In Canada, similar evaluations of telemedicine for cost and travel reductions have been made. This is particularly significant given Cloutier-Fisher et al.’s (2006) assessment that avoidable hospitalization rates are consistently higher in rural as compared to urban communities [20]. In particular, two studies focusing on the use of telemedicine for rural and remote communities in Northern Canada support the potential of telehealth to reduce the expenses of travel at both an individual level as well as at a systems-level [21, 22]. In terms of quality, an Australian study compared the diagnoses and treatment plans made during video conference appointments to in-person consultations for paediatric ENT surgery and found that the diagnosis was the same in 99% of cases (67 out of 68) and surgical management treatment plans were the same in 93% of cases (63 out of 68) [23]. Together this growing body of evidence suggests that telemedicine has the potential to deliver a cost-effective healthcare solution to rural communities by reducing the need for patient travel. However, it is also important to consider potential barriers to implementing telehealth, including inadequate local infrastructure in rural and remote communities (such as electricity and reliable internet) [10]. So far, one of the fundamental consequences precipitated by lack of access – the financial impact resulting from the need to travel to receive care – remains underexplored in a North American Context.

The consequences of challenges in accessing health care for rural residents range from increased stress incurred, above and beyond the inherent stress that accompanies a health crisis or event, to abandoning care altogether. Both of these responses have health consequences to the individual but also for the health care system itself as ‘upstream’ care reduces more advanced morbidity. To optimize access to health care for rural residents, it is essential to understand barriers to such care. The objective of this survey, administered in British Columbia (BC), Canada, was to estimate self-reported OOP costs for rural patients associated with accessing health care away from their home communities to move towards a comprehensive understanding of the costs of diminished rural health services. Along with direct costs, we have prioritized indirect cost estimates (e.g., loss of income due to time away from work or costs associated with escort travel) to present the best approximation of burden. Through this, effective health policy recommendations can be made to improve access to health care and thereby reduce rural disparities.

## Methods

### Study design

The OOP Costs Questionnaire was an online retrospective survey administered to rural BC patients who traveled to receive healthcare within the previous 2 years. The questionnaire, which ran from November 15th 2019 until March 292,020, was designed to accurately estimate the OOP spending of respondents as well as to evaluate their qualitative experiences, including patient stress and mobility issues. The choice of a retrospective survey was informed by both the associated difficulty of capturing all rural patient travel-costs across such an expansive area (rural BC), as well as the urgency for these rural patient voices to be heard. Recruitment relied on convenience sampling from participating referral centres via doctors and nurses making the survey available to patients, as well as from ads in local newspapers and on social media.

Eligibility criteria included: (i) rural citizens who were living in one of BC’s Rural Practice Subsidiary Agreement^1^ communities for at least 6 months prior to participating in the survey, (ii) at least 19 years of age, and (iii) traveled from their community to access care (or escort someone who needed care) within the previous 2 years.

This study was co-funded by the Health Economics Simulation Modelling Methods Cluster, BC SUPPORT Unit and the Joint Standing Committee on Rural Issues, through the larger context of the Rural Surgical and Obstetrical Networks program, which works to stabilize and enhance surgical and obstetrical services in rural communities across BC. The study was approved by the Behavioral Research Ethics Board at the University of British Columbia (Certificate Number H19–00445).

### Patient partner involvement

The survey instrument design was guided by four citizen-patient partners with personal experience traveling to seek healthcare, from communities representing a diversity of geography, size, and distance to the nearest referral health centres. These patient partners were selected through BC’s Patient Voices Network, a BC Patient Safety Quality Council initiative “linking patients, families and caregivers with health care partners who are seeking to engage the patient voices in their efforts to improve quality of care” [25]. Patient partners met monthly with the research team by teleconference during the development of the survey, as well as during the recruitment of participants and analysis of the data. Patient-partner perspectives were crucial in the development of the questionnaire, which aimed to determine the health priorities of rural citizen-patients and communities [26].

The survey consisted of 68 closed questions (Likert scale/yes-no/quantitative) and three open-ended questions, specifically targeted towards rural surgical patients. The instrument was pilot-tested in a small study sample (*n* = 56; ~ 15% of the total sample) from November 16 – December 4, 2019. Analysis of pilot-phase data alongside patient partners revealed the need to capture more broadly the costs incurred when traveling to access other forms of health care beyond surgery. We expanded the inclusion criteria to assess the costs incurred to access any health care covered by the provincial Medical Services Plan in BC, including both surgical and specialist care as well short-term disease management and chronic care management. The Medical Services Plan is the provincial health insurance program for BC residents and covers health care benefits including required medical services, diagnostic services, and some supplementary benefits [27]. The updated version of the survey was available on December 4th, 2019.

### Measures

The survey included questions about respondent demographics such as age-bracket, gender, ethnicity and household income-bracket, as well as about OOP cost estimation, patient stress, distance traveled and patient personal experience. Response options depended on the question type, which varied from numerical, categorical, Likert scale and open-ended comments. Skip-logic was implemented for multiple survey sections. Respondents were asked to report on their most recent health-care event (e.g., a surgical procedure, and/or cancer care) that required travel at most 2-years previous to filling out the survey. Some respondents included trips for multiple issues in one survey response, as travel was likely required for more than one condition/treatment at the same time.

**Fig. 1:**
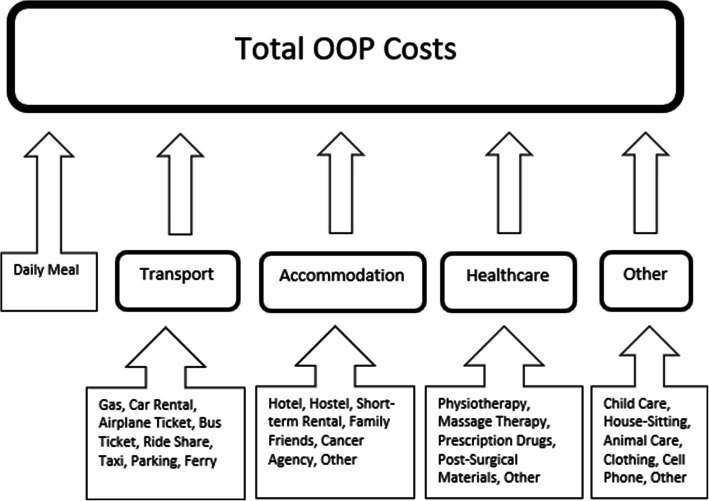
Cost categories and individual costs that comprise the Total OOP Cost estimate for each respondent

Total OOP cost estimates for each respondent were obtained as the sum of five cost-categories: (1) transport; (2) accommodation; (3) healthcare; (4) other; and (5) daily meal costs. Individual OOP expenses were grouped into these cost-categories to aid in interpretation of the survey results. For example, spending on *Car Rentals, Gasoline, Bus Tickets, Ferries, Taxi, Ride share* and *Parking,* were summed to obtain the *total transport cost* for each respondent. Likewise, aggregate amounts such as *total accommodation cost* or *total healthcare cost* were evaluated for each respondent. See Fig. 1 for a breakdown of these cost-categories.

For each kind of *transport* or *accommodation* related OOP expense, e.g. *filling up gas for a vehicle* or *paying for a hotel room*, respondents were asked to estimate their average cost for that expense, as well as the number of times they had to make that expense when traveling to receive healthcare over the previous 2 years. These estimates were multiplied to obtain the respondents’ total spending for that expense type. This was done to assist the respondent in providing a reasonable estimate for each expense. For all *healthcare costs* (e.g., prescription drugs, massage therapy)*,* respondents were asked about their overall spending for that cost type as well as the percent reimbursement. Total healthcare costs were obtained by subtracting the amount reimbursed from the overall amount. Otherwise, for all other related OOP expenses, respondents were simply asked to estimate their total expenditure.

In addition to the five cost categories used to determine *total OOP cost*, respondents were asked about co-traveler accommodation and co-traveler transport expenses, as well as about lost-wages due to travel. Other numerical questions included estimates of distance traveled to receive care (km), and Likert-scale measures of patient stress (1–10) before, after and during the hospital visit, as well as overall stress.

Except for demographic questions, mean and standard deviations of responses to each survey question were calculated using only those respondents who answered the question. For *transport* and *accommodation* expenses, means and standard deviations were calculated using only those respondents who answered both questions (number of purchases and average dollar amount per purchase). Six respondents reported very high individual transport or accommodation costs and were removed from all quantitative analyses to ensure that outliers did not significantly affect findings. All costs are reported in Canadian Dollars as of 2020.

### Data analyses

Only respondents who completed and submitted the survey were included in the analysis. Demographic data for non-completers was unavailable. The quality of the sample was assessed by comparing demographic distributions such as age, ethnicity, location, and household income, to those of the target population. Means and standard deviations were calculated for all numerical questions, as well as for all cost-categories, using only the total number of respondents for the response/ cost-category. For each cost-category, differences in total cost amounts between different income brackets were tested by one-way ANOVA F-tests comparing the mean *total amount spent* across different *reported income-brackets*. One-way ANOVA was also performed to assess bivariate relationships between distance traveled, transport costs, out of pocket costs, and patient stress. A summary of *Healthcare* and *Other* costs contributing to *total amount spent* can be found in Appendix 4 Tables 7 and 8. Analysis was conducted in R [28] and figures were produced using the package ggplot2 [29].

## Results

### Respondent characteristics

**Fig. 2:**
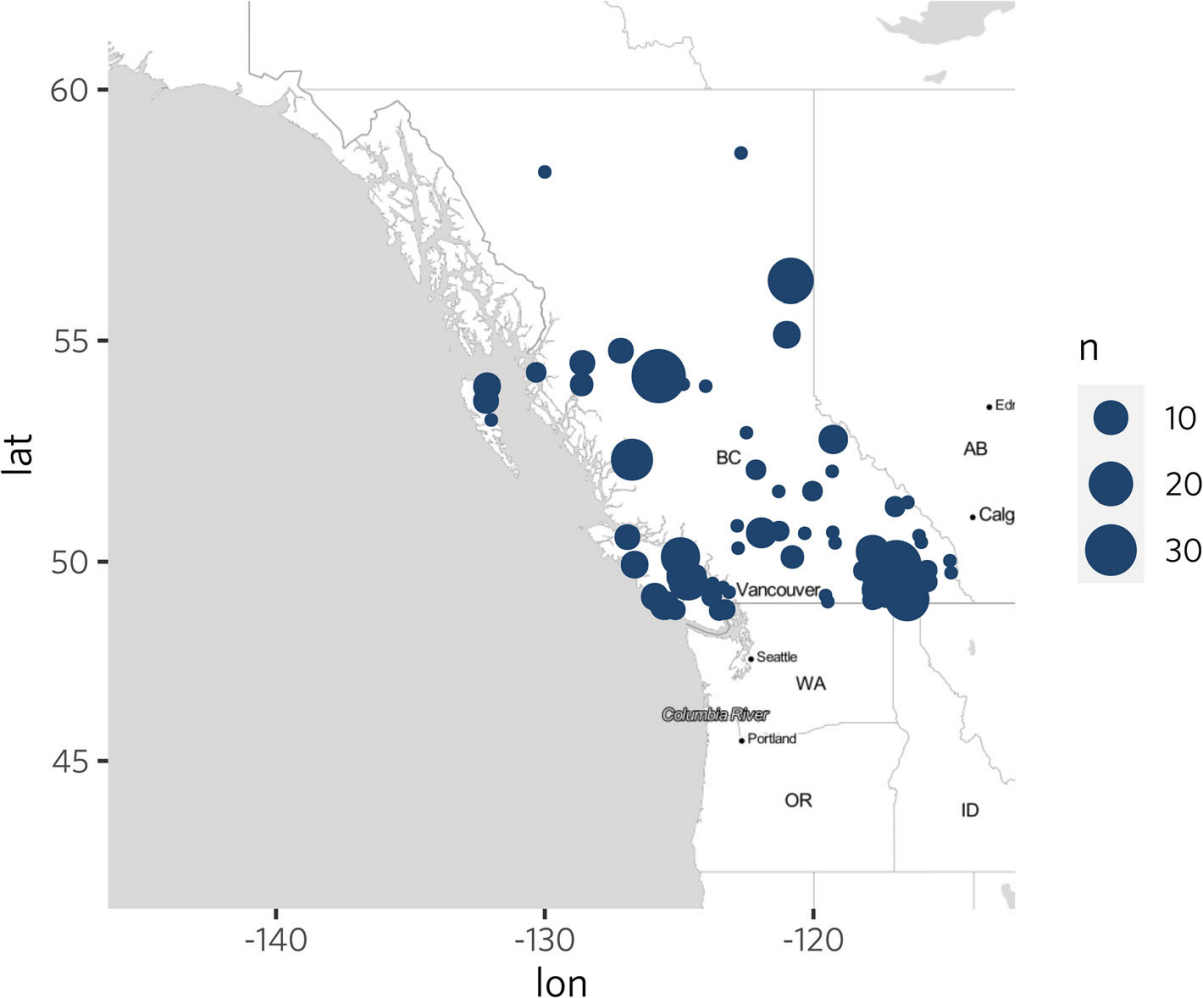
Home communities of survey respondents. This map was generated using R [28] packages ggplot2 [29] and ggmap [30]. Map tiles are by Stamen Design, under a Creative Commons Attribution (CC BY 3.0) license. *The size of the dot (“n”) corresponds to the number of respondents from each rural community*

A total of 381 rural citizens participated in this survey across rural BC; this included 56 respondents from the pilot test sample. The spatial distribution of respondents (Fig. 2) is shown and is visually comparable to a population density map of British Columbia. There were 65 respondents who completed the survey but were removed due to them having not answered most (90%) questions.

The average age of respondents at the time of the survey, approximated from their reported age-bracket, was 56 years (SD: 18 years). Of the 381 respondents, 87% were white (*n* = 333), 67% were female (*n* = 257) and 31% were male (*n* = 120). Many respondents had multiple reasons to travel for healthcare, with 51% traveling for surgical care (*n* = 193), 59% for specialist care (*n* = 226), 5% for chronic care management (*n* = 20) and 7% for short-term disease management (*n* = 26).

### OOP cost estimation

Average costs for different cost categories, as well as for *Total OOP* cost can be seen in Table 1. Excluding Co-Traveler costs, the largest cost category, both in terms of overall average cost ($856 CAD) and number of respondents included (346) was *Total Transport*, followed by *Healthcare* and then *Accommodation*. None of the cost-categories, including *Total OOP* costs, exhibited significantly different averages between household income brackets, indicating that there was likely not a strong relationship between OOP spending and household income.

**Table 1:** Summary of OOP Healthcare costs. Respondents were asked directly about their total OOP *Healthcare* spending for five categories, which are summed to obtain the *Total Healthcare cost* (see Table 3). The mean and standard deviation are provided for each category, as well as *p*-values obtained from a one-way ANOVA F-tests comparing the mean costs across different *reported income-brackets*

Cost-category	# of Respondents	% of Total	Total Spent $ Average (SD)
Total OOP	377	99	2044 (3094)
Total Transport	346	91	856 (1429)
Total Co-Traveler Transport	51	13	1077 (3678)
Total Accommodation	221	58	674 (983)
Total Co-Traveler Accommodation	96	25	862 (1570)
Daily Meal	301	79	387 (646)
Total Healthcare	233	61	694 (2236)
Total Other	118	31	384 (1007)

### Distance traveled and transportation costs

**Fig. 3:**
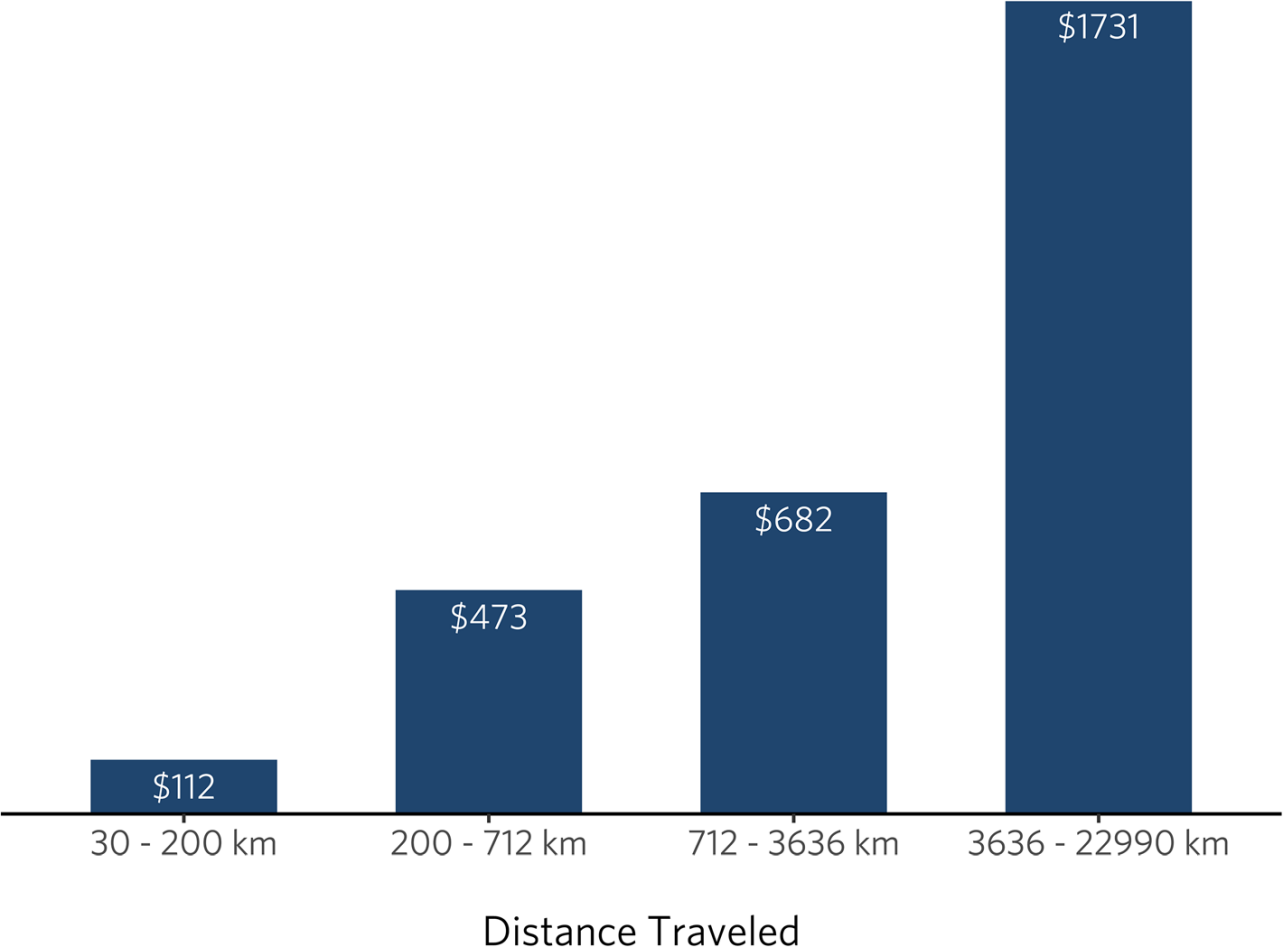
Average total out-of-pocket transport costs by distance traveled

Overall, the average transport cost per person was $856, and the average distance traveled per person to receive care was 1966 km. Among respondents who reported having *pre*-operative visit(s) for surgery (*n* = 167), most (87%) traveled outside of their community for their visit(s). This was also the case for respondents reporting *post*-operative visit(s) (*n* = 95) - 88% had to travel for their visit(s). Figure 3 illustrates the transport cost associated with the distance traveled.

**Fig. 4:**
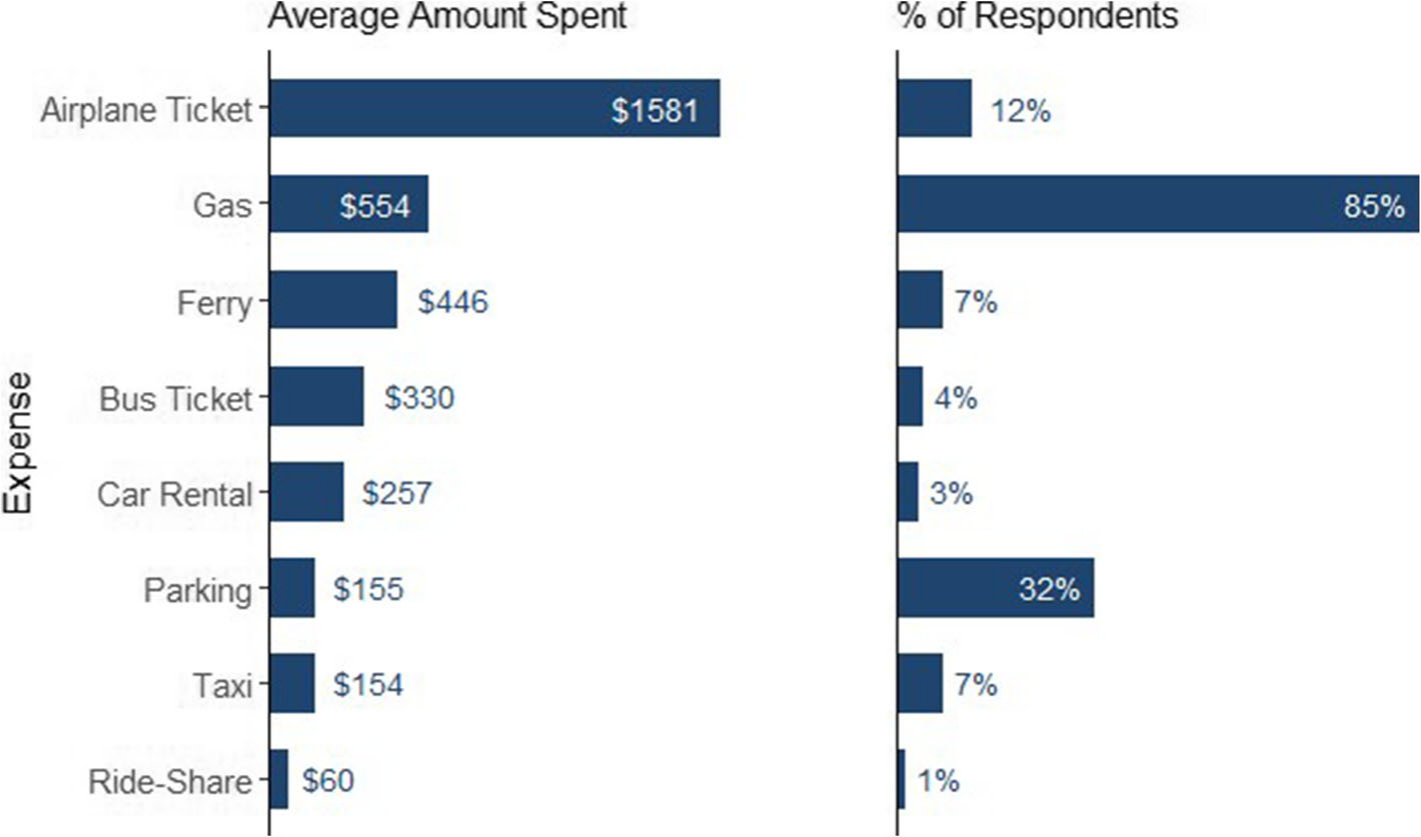
Average transportation costs by expense category for all respondents

Airplane tickets were the most expensive type of transportation and cost on average $1581. The most common type of transportation expense was gas, with 85% of respondents reporting this expense. Figure 4 and Table 2 provide more information on transportation costs.

**Table 2:** Summary of OOP transport costs*.* For each respondent, the *total amount spent* is calculated by multiplying the *reported average cost per purchase* by the *reported purchase frequency* for that specific cost category. The means and standard deviations are provided for the *total amount spent*, the *reported average cost per purchase*, and the *reported purchase frequency,* and are calculated using only the total number of respondents for the cost-category

Cost-category	# of Respondents	% of Total	Per Purchase $ Average (s.d)	Purchase Frequency Average (s.d)	Total Amount $ Average (s.d)
Gas	324	85	115 (132)	6 (14)	554 (1117)
Car Rental	10	3	162 (130)	2 (2)	257 (182)
Airplane Ticket	47	12	751 (418)	2 (1)	1581 (1393)
Bus Ticket	17	4	70 (69)	10 (22)	330 (626)
Ride Share	2	1	30 (14)	2 (0)	60 (28)
Taxi	25	7	44 (30)	6 (14)	154 (200)
Parking	122	32	20 (31)	9 (25)	155 (471)
Ferry	25	7	102 (95)	5 (4)	446 (484)

### Accommodation costs

More than half of survey respondents (58%) reported paying for accommodation. **These costs averaged $674 per person** and represented the second most expensive kind of OOP spending. Half of the respondents reported hotel expenses, which was also the most expensive type of accommodation. Although BC Cancer Agency offers subsidized housing for patients, a high average number of nights resulted in the highest average total costs for this accommodation type ($2205). Table 3 provides more information on accommodation costs.

**Table 3:** Summary of OOP accommodation costs*. For* each respondent, the *total amount spent* is calculated by multiplying the *reported average cost per night* by the *reported number of nights* for that specific cost category. The means and standard deviations are provided for the *total amount spent*, the *reported average cost per night*, and the *reported number of nights,* and are calculated using only the total number of respondents for the cost-category

Cost-category	# of Respondents	% of Total	Per Night $ Average (s.d)	# of Nights Average (s.d)	Total Amount $ Average (s.d)
Hotel	185	49	145 (54)	5 (6)	640 (829)
Hostel	4	1	20 (8)	6 (7)	142 (138)
Cancer Agency Accommodation	6	2	63 (23)	35 (60)	1780 (3029)
Family or Friend	25	7	55 (35)	10 (15)	344 (487)
Short-term Rental	12	3	103 (25)	7 (7)	715 (760)
Other	9	2	41 (30)	8 (9)	223 (274)

### Co-traveler out-of-pocket costs

**Fig. 5:**
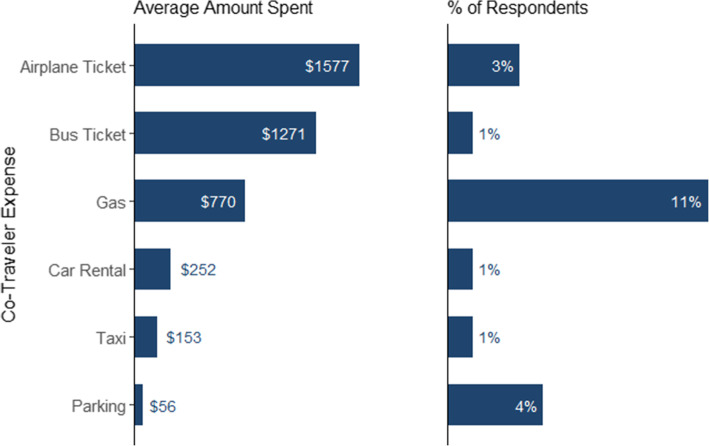
Average transportation expenses for co-travelers by category

Family members or friends accompanying patients also incurred significant OOP costs. Thirty-one percent of respondents reported separate co-traveler transport and/or accommodation expenses: 13% reported co-traveler transport costs, averaging $1077 per person and 25% reported co-traveler accommodation costs, averaging $862 per person. Refer to Appendix 1 Fig. 5 for more information on co-traveler transportation, and Appendix 2 Tables 5 and 6 for detailed information on co-traveler transport and accommodation expenses.

Most respondents (80%) traveled with someone who was not a health care professional. A spouse was the most common travel companion, followed by a child. While in the community of care, 18% of respondents had someone other than a co-traveler visit them. In total, 85% of respondents had a co-traveler and/or a visitor.

### System-level support for out-of-pocket costs

Only 14% of respondents reported having had some of their OOP transport and/or accommodation costs covered by organizations like the BC Travel Assistance Program or the First Nations Health Authority, the body responsible for the administration of health programs and services for B.C. First Nations [31]. Of the 53 people who received transport assistance, 37 did so through the BC Travel Assistance Program (mostly for ferry tickets), and five through the First Nations Health Authority. Only six respondents (2%) reported having received financial support for out-of-pocket accommodation spending, and five of these six respondents also reported assistance with transport costs.

### Lost wages

For many respondents, time spent away from home meant lost wages. When asked whether they had to take unpaid time off work to receive care, 93 respondents said yes and 56 said no (the remaining did not respond to this question). Those who lost wages missed an average of 17 workdays and an average of $2276 in personal income.

### Patient stress

Respondents were asked to rank their stress on a scale of 1–10 for their most recent health care visit (before, during, after, and overall) where 0 indicates no stress/anxiety and 10 indicates the worst imaginable stress/anxiety. The 360 people who responded to the question on overall stress reported an average stress level of 5.6 with a standard deviation of 2.7.

In a one-way ANOVA F-test comparing household income-bracket with pre-visit reported stress (Likert scale 1–10), a *p*-value of 0.016 was observed, meaning it is unlikely that income-bracket and pre-visit reported stress are unrelated. The negative correlation between reported stress and household income-bracket is shown by the average reported stress averages for each household income bracket (see Table 4), where it is clear that lower income respondents report higher average stress both before the visit and overall.

**Table 4:** Summary of responses to questions regarding patient stress provided for each income bracket, as well as for the overall data. Also provided are *p*-values from one way ANOVA F-tests for the differences in mean reported stress between household income-brackets. Not all respondents included in the total column are represented by the remaining columns, as only 331 (87%) respondents provided their income bracket

		Total	< $20,000	$20,000 - $39,999	$40,000 - $59,999	$60,000 - $79,999	$80,000 <
Pre-visit Stress	*p* = **0.016**						
	n	357	31	73	53	70	85
	mean	5.75	5.61	6.14	6.06	4.6	5.68
	sd	2.92	3.35	2.83	2.83	3.13	2.5
During Visit	*p* = 0.506						
	n	364	33	76	57	67	85
	mean	6.30	6.24	6.16	6.68	5.79	6.09
	sd	2.78	3.27	2.95	2.32	2.93	2.50
Post-visit Stress	*p* = 0.183						
	n	340	28	72	55	60	78
	mean	4.67	5.21	4.92	4.96	4.15	4.15
	sd	2.88	2.90	2.89	3.12	2.88	2.76
Overall Stress	*p* = 0.207						
	n	360	30	75	58	65	83
	mean	5.58	6.13	5.61	5.76	4.92	5.18
	sd	2.70	3.04	2.80	2.66	2.72	2.48

**Fig. 6:**
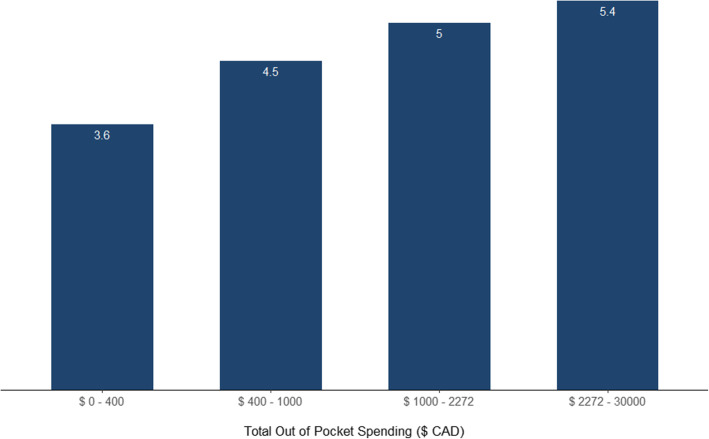
Average reported patient stress (scale 0–10) versus out-of-pocket spending

**Fig. 7:**
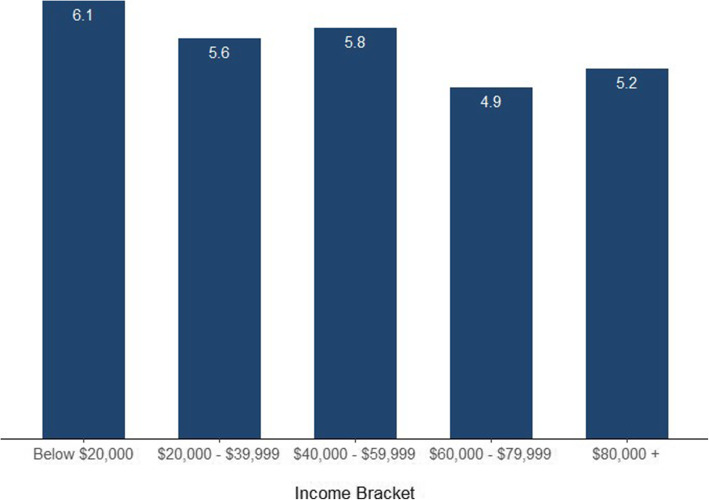
Average reported overall patient stress (scale 0–10) versus household income

Overall, the stress level was seemingly unaffected by whether or not someone received financial assistance. See Appendix 3 Figs. 6 and 7 for reported levels of stress by amount spent and income categories. The burden of traveling for care was particularly significant for maternity patients. Twenty-six percent of respondents reported that they were the caregiver of a child or other dependent. About half (52%) of these respondents with dependent(s) had to arrange for someone to care for their dependent(s) while they traveled to access care. Patient stress is discussed further in qualitative findings, below.

### Qualitative findings

Responses to three open-ended questions touched on multiple, intertwined themes including: challenges with transportation; the psychosocial impact of travel; the physical impact of travel; and delayed or diminished care-seeking. Each theme is described in detail below.

#### Challenges with transportation

Aside from financial costs, participants expressed that having to arrange and undertake transport was the most difficult part of leaving their home community to access care. Many participants commented on transportation difficulties in relation to their particular geographic environment. For example, many participants discussed the impact of winter road conditions on traveling to receive health care. Several participants recounted being involved in motor vehicle accidents. Other respondents commented that they had to delay care-seeking because they could not drive on dangerous winter roads and could not afford to travel. However, even for those patients who could afford to fly rather than drive, some still experienced issues getting back to their community due to winter weather conditions:

*“Even with being on disability and not having to deal with working around my work schedule, it is difficult to get out of my valley in the wintertime. Flights keep being cancelled and booked solid with no available seats. It’s great that the ticket is paid for but when you get stuck in [tertiary centre] for a week ‘cause of the flights being cancelled due to weather and no available seats the other expenses can really add up.”*

Other participants in water-bound communities discussed particular geographical challenges for arranging transportation to their health care appointments based on set ferry schedules.

Regardless of geographic location within the province, many respondents reported the expense of ‘wear and tear’ on their vehicles, such as flat tires and the need for early replacement of a car due to unexpected high mileage.

One of the most stark transport challenges for participants in this study, however, was securing transportation back home after an urgent event. As one participant noted,

*“I was taken by ambulance from [community] to [referral community], [then] had to find my way home. I was frail after a heart attack and it would have been difficult to take public transport.”*

#### Psychosocial impact of travel

Across a range of demographics, participants commented on the impact that having to travel for care had on their mental wellbeing. For many, dealing with a health condition had already caused some stress or anxiety, which was exacerbated by having to arrange and undertake travel. Unsurprisingly, those participants, who were not able to have a companion accompany them, expressed feeling particularly anxious or stressed as a result of having to leave their home communities while ill.

The psychosocial impact of traveling for care was particularly significant for maternity patients. Several participants in this study who had to leave their communities while pregnant to access pre-natal care or give birth, shared that the stress of having to pay for travel and accommodation may have contributed to their post-partum depression and/or anxiety. One participant from a remote community commented:

*“Expectant mothers in [my community] all have to leave the valley to have our babies. There are a number of medical visits before the delivery that we also have to leave the valley for. These include ultrasounds [and] specialist visits. The flights for these are covered, but not any other expenses and it gets expensive and stressful. Many families have to pay for a hotel while out waiting for the baby’s arrival. I was lucky and found a friend to stay with, but it is not overly comfortable staying with people in their home while waiting for my baby to arrive. You can never really relax. Then your support system [is] not there to support you.”*

Those respondents who did not express significant negative psycho-social consequences of travel all noted the presence of a strong support system. For example, one participant said:

*“I actually do not feel having my procedure outside of my home community had a negative impact on my recovery. However, I am very fortunate to have a caring spouse who took time off work to care for me. If she had not been able to be with me it would have made pre and post-surgery out of my community very inconvenient and likely would have impacted my recovery.”*

#### Time away from home and the physical impact of travel

In addition to the psychosocial stressors of not having social support, many participants expressed a range of other reasons why having to spend time away from home was difficult for them. For example, some participants commented on the challenges of eating out and staying in hotels with specific dietary or allergy-related concerns. Other participants commented more generally on the impact of having to travel on their physical recovery. One participant noted, “*As it [condition that required travel] was due to arthritis the driving was extremely hard on my muscles and joints*” while another observed, “*With chemo treatments I have no immune system to fight off germs*.” Several other participants affirmed the difficultly of having to travel directly after a hospital procedure. As one participant noted, *“…the most difficult procedure for me was the biopsy and I had to fly home with a bleeding and painful wound.”*

Spending time away from home was particularly difficult for families with young children. Challenges included having to miss school to attend their parents’ medical appointments and needing specific types of care from parents that made it difficult to be away from them. For example, one woman described the impact of an unexpected surgery on her husband and young child:

*“This was an unexpected emergency surgery that happened [the] same day symptoms presented themselves. My husband and son accompanied me to the hospital and when they decided I would require surgery and an overnight stay, my husband needed to head back home with our 10-month-old as he had not prepared for an overnight [stay]. I was also not able to breastfeed due to medications and we had no breast milk on hand. This meant they needed to make the 2-h trip back the next day to get me and then 2 h home again. Lots of driving for a small child.”*

#### Rural gaps

Many participants expressed that having to travel for certain types of care was expected as a rural resident. However, they also felt that there were some essential services that should be available in their local community but were lacking. Most notably, there was a perception that many rural communities are lacking an adequate number of family doctors, leading to an over-reliance on emergency services. As a result of the closure of the walk-in clinic in their rural community, one participant even commented that they felt they had no other choice but to pay for private care.

A second rural health gap described by some respondents was the lack of alternatives to in-person specialist visits, such as visiting specialists or opportunities for virtual care. This was perceived by some participants to be the result of inadequate systems planning. As one person commented:

*“[I] Travelled to a specialist appointment in [referral centre], and was required to stay overnight due to time of appointment. Information given at the appointment could easily have been conveyed by my GP in [home community] as it was not urgent. I could have saved the travel time, 2 days away from work, gas, hotel and food if this could have been done either through my regular doctor or even via skype or a health portal.”*

#### Delayed or diminished care seeking

Some participants commented that after considering the costs and impacts of travel, they delayed or diminished their health care seeking. One participant said, *“My child should be assessed for autism but the trip to Prince George is unaffordable.”* Delayed or diminished care seeking seemed to be more common among individuals who had to rely on others to take them to health care appointments. Others commented from the perspective of a family caregiver, noting the difficulty in ensuring access to recommended care:

*“I cannot take time off work to get my disabled mother to some recommended medical therapies that are not available in or near my home community.”*

Even the knowledge that a local doctor would likely refer the patient to a distant specialist prevented some individuals from seeking care in the first place:

*“I have not gone to the Dr. knowing that they would send me to a specialist far away and we couldn’t afford the costs at the time.”*

While many participants commented that they had to budget and plan for costs associated with traveling for health care services, some expressed that they would have to cancel or reschedule their appointments at the last minute due to unexpected inability to afford travel. One respondent noted, “*Postponed neurological appointments because I could not afford travel. Credit cards and credit line maxed out*” while another commented, “*[I] have had to cancel out of town medical appointments due to loss of wages and burden of finding child care.*”

## Discussion

We have estimated that rural patients traveling to seek healthcare spend an average of $2044 CAD in OOP costs per person per condition, broken down into several categories and sub-categories for rural patients traveling for healthcare, and we have documented the relevant personal experiences of these patients. Both the average amount spent on each category (transport, accommodation, etc.) as well the number of respondents having reporting expenses for each category help illustrate the scope of OOP spending. The additional impact of lost-wages and system-level support were also investigated. Finally, strong relationships were found between distance traveled and OOP spending, as well as between patient stress and household income-bracket.

Like all voluntary retrospective survey studies, we anticipate that BC residents who experienced greater difficulty in dealing with the financial and psychosocial burden of traveling for health care were more motivated to respond, thus potentially limiting the transferability of our findings to all rural BC residents. A more involved study design that includes random sampling with balanced treatment groups would be required to obtain more accurate estimates of OOP spending, and such a design would require population-size estimation (estimating the proportion of patients from each community seeking healthcare outside of their community). Future attempts to estimate the OOP spending of rural BC patients traveling to seek healthcare should therefore refer to population-level data to inform their sampling approach. In any case, the estimates obtained from our survey are reasonable and expected given each patient’s location and condition. A cursory comparison of our cohort demographics with that of the BC rural population showed that proportions of respondents from various income brackets and ethnic groups were generally consistent with those of the BC population, however, the age distribution of survey respondents differed from that of rural BC, with the average age for our cohort (53) being much higher than that of Rural BC (42).

Balancing these potential limitations, however, was the relatively high number of responses and geographical spread of respondents. Regardless, as this is the first rigorous collection and presentation of comprehensive OOP costs for rural residents traveling to access health care, we feel it provides useful information to an under-explored area of health care experiences.

At the time of pan-Canadian regionalization, Church and Baker suggested that Canada’s geography makes it difficult to achieve the economies of scale that make regionalization a functional model for more densely populated jurisdictions [32]. Specifically, they note,

“…all in all, regional populations in Canada might be too small to achieve any real economies of scale or to more generally affect coordination of health services”^2^

A Ministry of Health appointed Advisory Committee on Ontario’s Rural Health Hubs Framework found that insufficient public transportation was one characteristic of rural communities that limits access to care [33]. We have seen diminished support for rural transport through for-profit services across Canada (for example, through the withdrawal of Greyhound bus services) which exacerbated the growing issue of transport to specialist care in regional communities and, predictably, has disproportionately disadvantaged vulnerable populations with reduced social and financial capital. In this way, transport (as a proxy to access to care) has become a social determinant of health.

Patient-travel from rural and remote communities to larger centres is a key assumption of regionalized health care systems where patients benefit from regional specialist care. In many jurisdictions, this improves access to such care for most of the population, as they no longer need to travel to larger urban centres, thereby affecting the ‘closer to home’ advantage. For smaller rural and remote communities, however, regionalization can diminish local access to all, but primary care as regional procedural care must include the caseload of rural residents in order to maintain a viable case volume for specialist call groups. From a systems perspective, this may be an appropriate cost-benefit calculation with increased rural patient travel being a necessary by-product. There are however, productive ways of ameliorating the effect on rural residents through a reconceptualization of both patient travel and system supports for those instances when travel is necessary.

A consequence of the regionalization of health services that many jurisdictions in BC have undergone in the past two decades has been the attrition of specialists in low-volume communities in exchange for regional concentrations. Although access to care is still prioritized, the default mechanism of achieving this (in non-urgent situations) has been through patient-initiated travel. This is not the case, however, in many instances where regional specialists provide clinical care in smaller communities through regularly scheduled outreach visits. This is usually contingent on having enough accrued patient volume to justify the travel. When this is not possible, optimizing the potential of virtual care, either with or without the involvement of local care providers, also acts to lessen the burden of travel for rural patients. This may involve virtual visits between a specialist and rural patient supported by a local care provider or direct specialist-to-patient care. We have recently seen the capacity for health system adaptation for increased virtual care in the face of COVID-19; instilling the infrastructural resources and workflow patterns into rural and referral communities to support the expectation of virtual care where possible will create a legacy for this adaptation. When framed within these opportunities to reduce non-urgent patient travel, we can recognize the value of a paradigm shift where instead of being the first recourse to access to care, patient travel becomes the last resort.

Within this framework, the health system could, for example, immediately reduce the need for travel for pre-operative care. In this study, 87% of respondents who reported pre-operative visits reported having to travel for such care (and 88% post operatively) despite respondents’ perception of the lack of urgency for in-person visits. Preoperative care could reasonably be offered through virtual care in many circumstances or, in some instances, involving local care providers in a tripartite model with the patient and specialist if hands-on care is required.

There are other system-level solutions to minimize the ‘rural tax’ on patients in accessing health care, such as supported accommodation in regional referral centres. In this study, respondents paid an average of $674 in accommodation costs. There are already models in place providing subsidized accommodation to defray such costs in cases like cancer care or pediatric emergencies that we could learn from.

Further, the importance of social support in optimizing health outcomes must not be underestimated, nor the costs associated with this support. In this study, 85% of respondents were accompanied by and/or had visitors in the referral centre. This additional expense is often covered by the patient themselves, particularly in situations where accompanied travel post-visit is essential. In considering a holistic view of wellness, the mitigating influence of social support would be wise to consider not just to reduce system costs, but as a compassionate counterpoint to frequent criticism of ‘depersonalized health care’ [34, 35]. In instances where patients struggle to afford travel costs for themselves, let alone escorts, the question of whose responsibility it is to enable access to necessary health care remains.

To our knowledge, this is the first primary research study to systematically document the financial consequences of traveling for care for rural residents both in Canada and other comparable jurisdictions and, as such, provides important information for health care planners. A broader societal perspective of costs, including costs that are transferred to individuals and families, is essential to include in health care planning and decision-making, especially given that the impact of OOP cost expenses are most strongly felt by those who lack financial and social resources. A broad view of cost accounting also includes considering less tangible costs, such as increased stress and anxiety that occur alongside the stress of the medical event. This may be due to not only financial worries, but also as a result of losing support networks of family and friends when having to travel. If the time out of the community is extended, then there is also disruption to usual routines, which is particularly difficult for caregivers including those with young children. Although we acknowledge that “not everything that can be counted counts and not everything that counts can be counted,” through the rich descriptive comments provided by survey respondents, we can start to better understand the consequences of traveling for care.

## Conclusion

The results of this survey provide a starting place for discussions on the role of public support for rural residents who need to travel for health care. Rigorously documenting costs associated with access to health care for rural residents shows, for the first time, the burden of travel and consequent financial barriers this presents for some citizens. This burden is difficult to appreciate by anyone who does not experience rural geography but is essential to consider in health care planning and in a regionalized health care system built on increasing availability of services to respond to population needs, leading to specialist concentration in larger centres. It is our hope that accommodation can be made for patients that need to travel from rural communities, such as later appointment times to allow same-day travel and flexibility to change appointments without penalty due to travel-related obstacles (e.g., treacherous road conditions in winter). This data provides a helpful starting point for discussions on how to weave a safety net to ensure rural citizen-patients can access care outside their communities. These discussions must involve key stakeholders from rural communities but also regional representatives and government ministries entrusted with ensuring appropriate access to care, transportation and social development. Bringing the right group together in jurisdictions challenged with patient travel for non-acute health care will provide a starting place for developing a system response to ensure all residents have access to the health care they require, without financial barriers.

## Data Availability

The datasets used and analysed during the current study are available from the corresponding author on reasonable request.

## Appendix 2

### Co-traveler OOP costs

**Table 5:** Summary of co-traveler OOP accommodation costs. For each respondent, the *total amount spent* is calculated by multiplying the *reported average cost per night* by the *reported number of nights* for that specific cost category. The means and standard deviations are provided for the *total amount spent*, the *reported average cost per night*, and the *reported number of nights,* and are calculated using only the total number of respondents for the cost-category

Cost-category	# of Respondents	% of Total	Per Night $ Average (s.d)	# of Nights Average (s.d)	Total Amount $ Average (s.d)
Hotel	85	22	140 (41)	5 (5)	657 (773)
Hostel	2	1	40 (42)	3 (3)	180 (240)
Cancer Agency Accommodation	2	1	50 (1)	76 (105)	3850 (5374)
Family or Friend	9	2	51 (48)	14 (32)	1214 (3295)
Short-term Rental	4	1	144 (42)	10 (10)	1674 (2224)
Other	1	0	60 (NA)	21 (NA)	1260 (NA)

**Table 6:** Summary of co-traveler OOP transport costs*.* For each respondent, the *total amount spent* is calculated by multiplying the *reported average cost per purchase* by the *reported purchase frequency* for that specific cost category. The means and standard deviations are provided for the *total amount spent*, the *reported average cost per purchase*, and the *reported purchase frequency,* and are calculated using only the total number of respondents for the cost-category

Cost-category	# of Respondents	% of Total	Per Purchase $ Average (s.d)	Purchase Frequency Average (s.d)	Total Amount $ Average (s.d)
Gas	41	11	70 (46)	7 (23)	770 (3485)
Car Rental	3	1	185 (26)	1 (1)	252 (130)
Airplane Ticket	11	3	577 (324)	3 (2)	1577 (1188)
Bus Ticket	3	1	36 (27)	27 (30)	1271 (2017)
Ride Share	0	0	NA (NA)	NA (NA)	NA (NA)
Taxi	4	1	56 (33)	4 (3)	153 (109)
Parking	15	4	17 (15)	4 (3)	56 (39)

## **Appendix 4**

### OOP Healthcare and Other Costs

**Table 7:** Summary of OOP Healthcare costs*.* Respondents were asked directly about their total OOP *Healthcare* spending for five categories, which are summed to obtain the *Total Healthcare cost* (see Table 1). The mean and standard deviation are provided for each category, as well as *p*-values obtained from a one-way ANOVA F-tests comparing the mean costs across different *reported income-brackets*

Cost-category	# of Respondents	% of Total	Total Spent $ Average (s.d)	*p*-value (Income)
Physiotherapy	45	12	320 (527)	0.44
Massage Therapy	31	8	268 (353)	0.51
Prescription Drugs	183	48	287 (646)	0.88
Post-Surgical Materials	140	37	169 (508)	0.76
Other Healthcare Costs	44	12	1427 (4606)	0.53

**Table 8:** Summary of “Other” OOP costs*.* Respondents were asked directly about their total *Other* OOP spending for six categories, which are summed to obtain the *Total Other cost* (see Table 1). The mean and standard deviation are provided for each category, as well as *p*-values obtained from a one-way ANOVA F-tests comparing the mean costs across different *reported income-brackets*

Cost-category	# of Respondents	% of Total	Total Spent $ Average (s.d)	*p*-value (Income)
Child Care	13	3	1017 (2719)	0.47
House-Sitting	33	9	244 (255)	0.88
Animal Care	64	17	166 (183)	0.29
Clothing	15	4	129 (117)	0.72
Cell Phone	46	12	99 (164)	0.41
Other	19	5	365 (600)	0.95

## Abbreviation

OOP: Out of pocket

## Footnotes

1 The Rural Practice Subsidiary Agreement aims to enhance physician services and patient care in rural and remote areas of BC [24]. Communities must receive a minimum number of ‘isolation points’ to be eligible for programs under this agreement, which is between the provincial government, Doctors of BC, and the Medical Services Commission. Rural communities across BC are identified by degrees of isolation.
